# Two Cases Showing That Cilostazol Administration Leads to an Increase in Cerebral Blood Flow and Has a Positive Effect on Rehabilitation

**DOI:** 10.7759/cureus.56376

**Published:** 2024-03-18

**Authors:** Shuji Matsumoto, Rintaro Ohama, Takashi Hoei, Ryuji Tojo, Toshihiro Nakamura

**Affiliations:** 1 Center for Medical Science, Ibaraki Prefectural University of Health Sciences, Ami, JPN; 2 Department of Rehabilitation and Physical Medicine, Kagoshima University Graduate School of Medical and Dental Sciences, Kagoshima City, JPN; 3 Department of Rehabilitation, Kagoshima University Hospital, Kagoshima City, JPN; 4 Department of Rehabilitation, Acras Central Hospital, Kagoshima City, JPN

**Keywords:** hemiplegia, outcome, rehabilitation, cerebral blood flow, cilostazol

## Abstract

Cilostazol is a drug that has both antiplatelet and vasodilatory effects. To examine the effects of cilostazol on cerebral blood flow and rehabilitation following stroke, cilostazol was administered to two patients with chronic atherothrombotic cerebral infarction. In both patients, cilostazol administration effectively increased cerebral blood flow and promoted rehabilitation. Therefore, cilostazol was considered to be a useful agent for improving the clinical condition of patients suffering from chronic cerebral infarction. Further clinical studies on the effective use of cilostazol for rehabilitation in stroke patients are needed.

## Introduction

Although the mortality rate as a result of stroke is declining, the incidence of stroke itself is increasing. As a result, the number of patients with chronic cerebral infarction is also rising, such that the clinical management of this condition is likely to become a major future health issue. The global number of deaths from stroke is projected to increase from 2.04 million to 3.29 million between 1990 and 2019 and to 4.9 million by 2030 [1].

There is now widespread evidence that antiplatelet drugs are an effective treatment for atherothrombotic cerebral infarction [2], but there are currently no indices as to what types of antiplatelet drugs are most effective or at what stage they should be administered. In addition to an antiplatelet effect [3] due to cyclic guanosine monophosphate (cGMP)-inhibited phosphodiesterase, cilostazol also reportedly has pleiotropic and vasodilatory effects [4,5], improves vascular endothelial function [6] and suppresses vascular smooth muscle growth [7]. Cilostazol reportedly enhances cerebral blood flow in cases of chronic cerebral infarction [8]. However, its effects on physiological functions, the performance of activities of daily living (ADL), and cognitive function have not been investigated previously.

We administered cilostazol to two patients with chronic atherothrombotic cerebral infarction and evaluated the effects of the drug on cerebral blood flow and rehabilitation.

## Case presentation

Case 1

**Figure 1:**
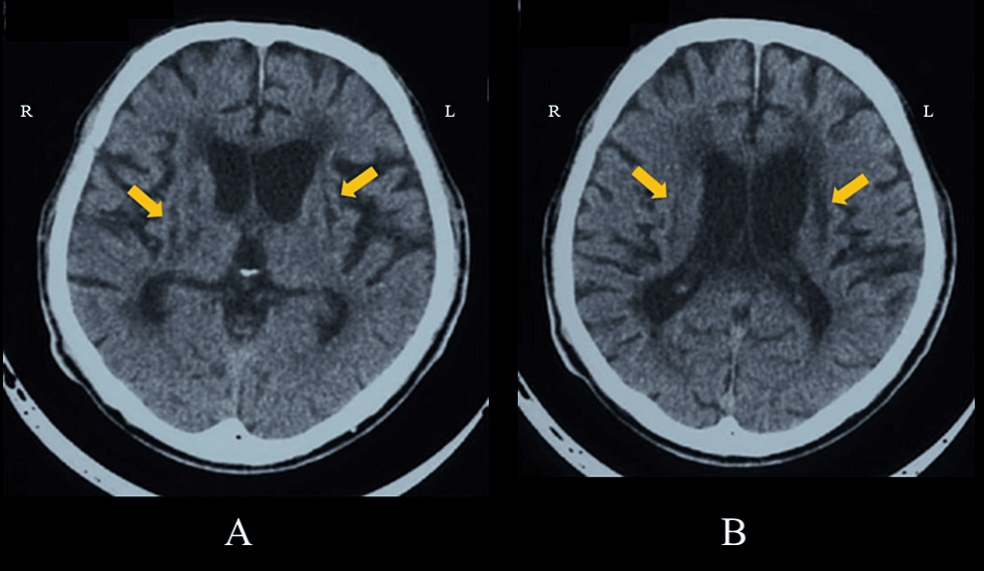
Brain computed tomography (CT) (Case 1). Brain CT revealing low-density areas in the bilateral basal ganglia, internal capsule, and corona radiata (arrow): (A) basal ganglia level slice view and (B) corona radiata level slice view. R: right side, L: left side.

Case 1 was a 56-year-old man whose chief complaint was difficulty walking and dysarthria. His past medical history was hypertension since the age of 46. His family history included a younger brother with subarachnoid hemorrhage and an older brother with hypertension. Current medical history includes left hemiplegia and gait disturbance, and a diagnosis of atherothrombotic cerebral infarction. Seven months after the onset of stroke, he was admitted to our hospital for rehabilitation. As a clinical condition at the time of initial presentation, left hemiplegia was classified as Brunnstrom stage (BRS) 3 in the upper extremity and fingers and 4 in the lower extremity. The Fugl-Meyer Assessment (FMA) motor score was 33 (maximum 66) for the upper extremity and 22 (maximum 34) for the lower extremity. For ADL, the patient’s Barthel Index (BI) score was 20/100. The patient’s cognitive function score according to the Mini-Mental State Examination (MMSE) was 18/30. Computed tomography (CT) of the brain revealed low-density areas in the bilateral basal ganglia, internal capsule, and corona radiata (Figure 1). Using a xenon-CT, blood flow was evaluated by standard slicing of the bilateral hemisphere, basal ganglia, thalamus, and cortex [9]. For the cerebral blood flow imaging, the regions of interest (ROI) were the right and left cerebral hemispheres, right and left putamen, right and left thalami, right and left frontal cortices, right and left temporal cortices, and right and left occipital cortices. The blood flow in the cerebral hemispheres was 19.7 and 18.0 mL/min/100 g in the right and left hemispheres, respectively.

**Figure 2:**
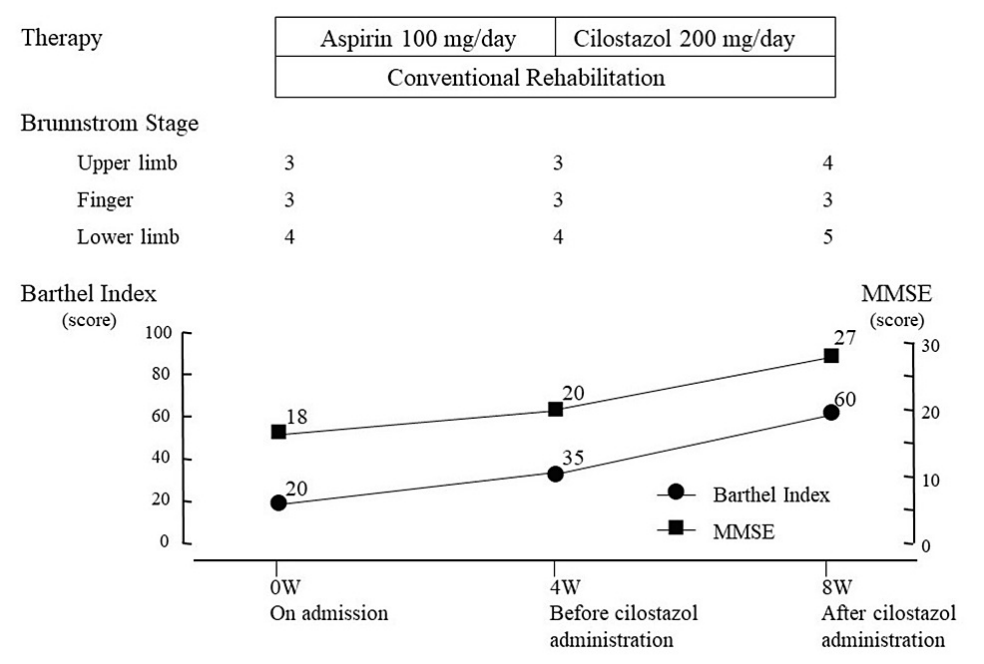
Clinical course of Case 1. MMSE: Mini-Mental State Examination.

The clinical course is shown in Figure 2. Case 1 was prescribed aspirin by a previous doctor in order to prevent the secondary occurrence of cerebral infarction. He also underwent a rehabilitation program as per the instructions of that doctor. For the first four weeks after admission, Case 1 received aspirin 100 mg/day and conventional rehabilitation consisting of physical therapy, occupational therapy, and speech therapy. After these four weeks, the antiplatelet medication was switched from aspirin to 200 mg/day of cilostazol, which was administered for a further four weeks. Five times a week, the subject underwent a conventional stroke rehabilitation program consisting of range of motion (ROM) exercises, muscle strengthening, basic activity training, gait and ADL training, and speech therapy as indicated. The administration of drugs that might influence cerebral blood flow was prohibited during the study period.

**Figure 3:**
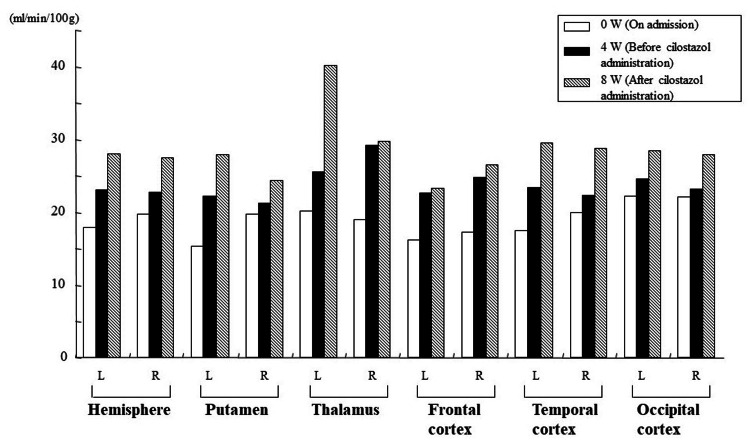
Cerebral blood flow (CBF) in each region of interest using xenon-CT (Case 1). CT: computed tomography.

Figure 3 shows the examination of cerebral blood flow of Case 1 using xenon-CT at three time points: at the time of hospitalization, after four weeks of hospitalization (before cilostazol administration), and after four weeks of cilostazol administration (eight weeks after hospitalization). Figure 3 shows that cerebral blood flow improved dramatically in all ROIs after cilostazol administration.

With regard to the patient's condition, mild improvement in left hemiplegia was achieved while on cilostazol throughout the clinical course. After four weeks, the BRS of the upper extremity, fingers, and lower extremity were 3, 3, and 4, respectively. After eight weeks, the BRSs of the upper extremity, fingers, and lower extremity changed to 4, 3, and 5, respectively. After four weeks, the FMA scores of the upper and lower extremities were 35 and 24, respectively, and after eight weeks, the FMA scores of the upper and lower extremities were 44 and 28, respectively. The BI score changed from 20 to 35 while on aspirin and from 35 to 60 while on cilostazol. The BI score changed from 35 to 60 during cilostazol administration, with much greater improvement (Figure 2). MMSE also showed a much greater improvement with cilostazol, with seven changes during cilostazol treatment compared to only two changes during aspirin treatment (Figure 2).

Case 2

**Figure 4:**
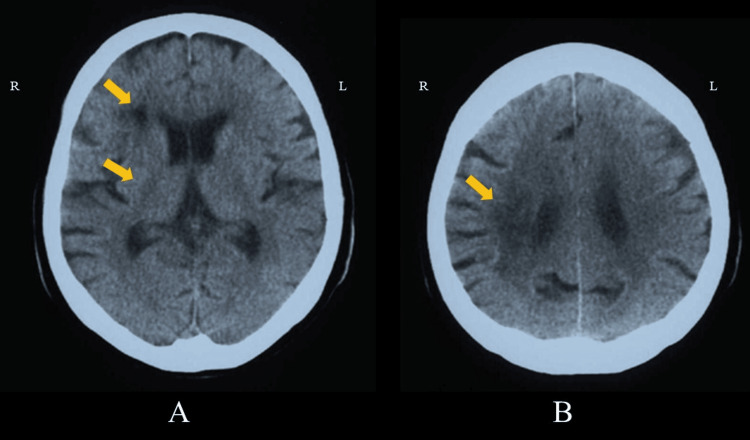
Brain CT (Case 2). Brain CT revealing a low-density area in the right middle cerebral artery territory, including putamen, corona radiata, and centrum semiovale (arrow): (A) basal ganglia level slice view and (B) semioval center level slice view. R: right side, L: left side, CT: computed tomography.

Case 2 was a 70-year-old woman whose chief complaint was difficulty with ambulation. Her past medical history includes a 20-year history of hypertension, and she has been taking oral medication since she was about 50 years old. There is no family history of hypertension. Her current medical condition was left hemiplegia and gait disturbance, and she was diagnosed with atherothrombotic cerebral infarction. She was admitted to our hospital for rehabilitation five months after the onset. On admission, the clinical condition of case 2 was left hemiplegia with Brunnstrom stage 4 upper extremity and fingers and stage 5 lower extremity. The FMA motor scores for Case 2 were 38 for the upper extremities and 27 for the lower extremities. For ADL, the patient’s BI score was 60/100. The cognitive function score, according to the MMSE, was 21/30. The brain CT (Figure 4) revealed a low-density area in the territory of the right middle cerebral artery, including the right putamen, corona radiata, and centrum semiovale. Cerebral blood flow was evaluated using a xenon-CT scan, and the same ROIs were used as described for Case 1. The cerebral hemispheric blood flow is 23.9 and 25.2 mL/min/100 g in the right and left hemispheres, respectively.

**Figure 5:**
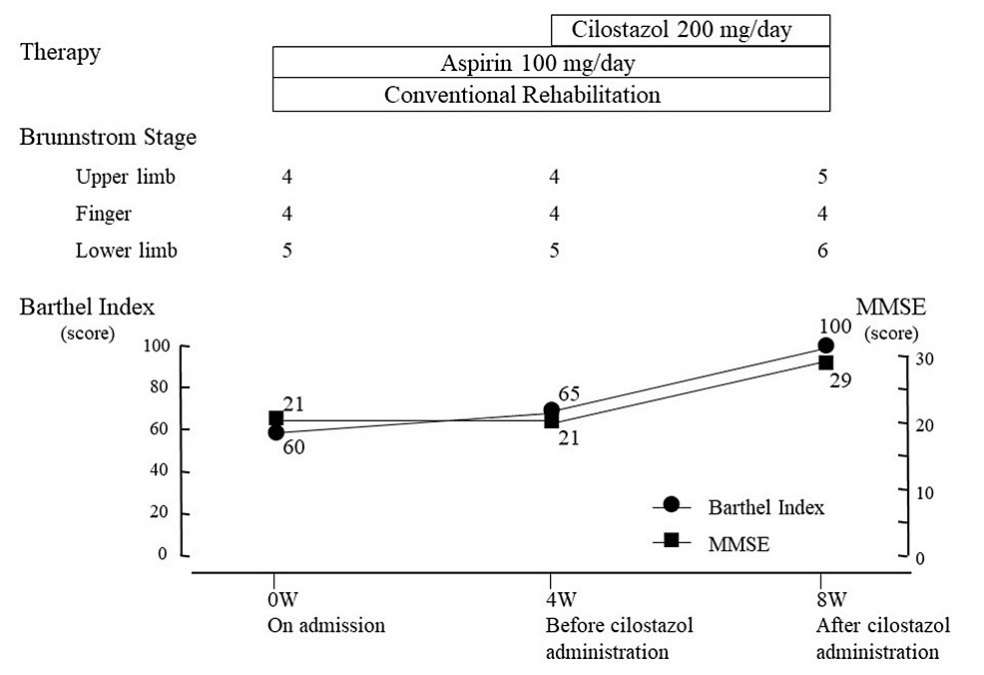
Clinical course of Case 2.

The clinical course is shown in Figure 5. Case 2 was prescribed aspirin by a previous doctor in order to prevent the secondary occurrence of cerebral infarction. She also underwent rehabilitation on the instructions of that doctor. For four weeks after hospital admission, Case 2 was treated with 100 mg/day of aspirin and conventional rehabilitation, comprising physical therapy, occupational therapy, and speech therapy. After four weeks, 200 mg/day of cilostazol was administered in addition to aspirin for a further four weeks as antiplatelet medication. Five times a week, the subject underwent a conventional stroke rehabilitation program, consisting of ROM exercises, muscle strengthening, basic activity training, gait and ADL training, and speech therapy as indicated. The administration of drugs that might influence cerebral blood flow was prohibited during the study period.

**Figure 6:**
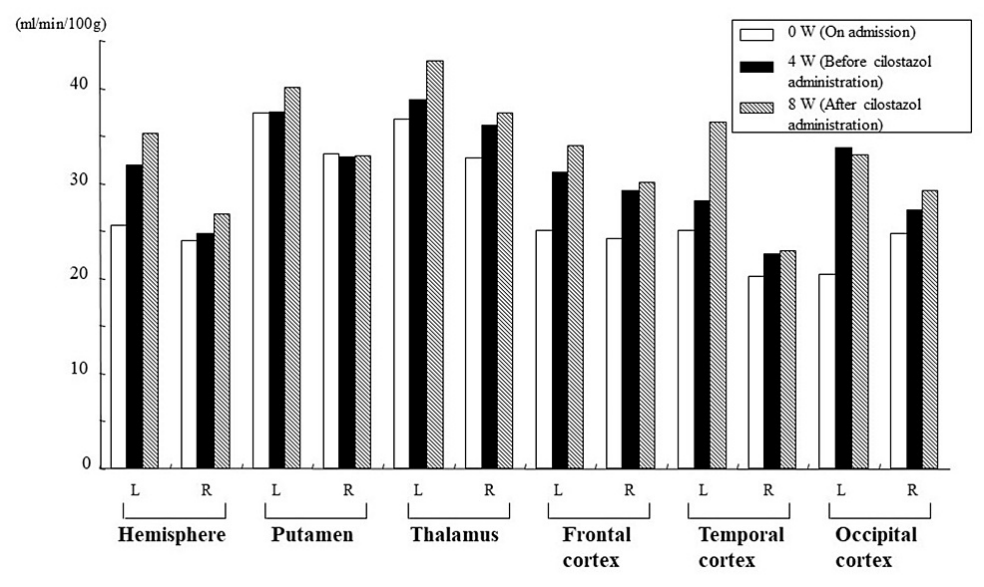
CBF in each region of interest using xenon-CT (Case 2). CBF: cerebral blood flow; CT: computed tomography.

Figure 6 shows the cerebral blood flow examination of Case 2 using the xenon-CT at three time points: at the time of hospitalization, after four weeks of hospitalization (before cilostazol administration), and after four weeks of cilostazol administration (eight weeks after hospitalization). The cerebral blood flow in each ROI shown in Figure 6 indicates that cilostazol administration improved cerebral blood flow in all ROIs except one.

After four weeks, BRS scores for the upper, hand, and lower extremities were unchanged at 4, 4, and 5, respectively; after eight weeks, BRS scores for the upper, hand, and lower extremities changed to 5, 4, and 6, respectively. After four weeks, the FMA scores for the upper and lower extremities were 39 and 27, respectively; after eight weeks, the FMA scores for the upper and lower extremities were 51 and 32, respectively. Rehabilitative evaluation of Case 2 showed greater improvement in left hemiplegia after administration of cilostazol. The change in BI was 5 before cilostazol administration and 35 after (Figure 5). MMSE was unchanged before cilostazol administration and changed by 8 after administration (Figure 5).

## Discussion

Cilostazol was well tolerated and no complications, side effects, or adverse events were noted during the eight-week study period. Neither of the subjects experienced discomfort before, during, or after the administration of cilostazol. The rehabilitation outcome measurements (i.e., BRS, FMA, BI, and MMSE) were also completed safely in both subjects. In both cases, cerebral blood flow improved with cilostazol administration, and rehabilitation outcomes were favorable.

The risk factors for cerebral infarction include high blood pressure, diabetes mellitus, hyperlipidemia, advanced age, smoking, and carotid stenosis. To prevent the recurrence of cerebral infarction, it is important to manage these risk factors; hence, aspirin is often prescribed as an antiplatelet drug. However, when aspirin alone is not sufficiently effective, especially in high-risk patients with a lacunar infarction [10] or carotid artery lesion [11], it is often either replaced with cilostazol or given in combination with cilostazol.

Cilostazol is an antiplatelet drug that, in contrast to aspirin, causes comparatively few hemorrhagic complications [10]. The randomized, controlled Chinese Acute Stroke Trial (CAST) showed that statistically, aspirin was useful for the treatment of acute cerebral infarction, although there was only a 0.6% difference from the placebo-administered group. However, incidences of hemorrhagic complications reportedly increased by 0.2% compared with those in the placebo group [12]. A meta-analysis of a clinical study of patients with chronic cerebral infarction also showed that there were significantly more adverse hemorrhagic reactions following treatment with aspirin than when a placebo was administered [13]. As an antiplatelet agent, cilostazol, in contrast to aspirin, has the advantage of relatively few bleeding complications [10]. A meta-analysis of clinical trials in stroke patients showed that cilostazol, alone or in combination with aspirin, significantly reduces recurrent stroke, post-stroke intracranial hemorrhage, and extracranial bleeding in patients with prior ischemic stroke compared with other antiplatelet therapies [14].

The Cilostazol Stroke Prevention Study (CSPS) [10] was a randomized, double-blind study of cerebral infarction recurrence rates among 1,052 patients in Japan who were administered either cilostazol or a placebo for one to six months after the onset of cerebral infarction. Cilostazol was shown to mitigate the risk of cerebral infarction by 41.7% compared to placebo. In a subanalysis in which patients were grouped according to the type of cerebral infarction, cilostazol significantly reduced the relative risk of lacunar infarction. For atherothrombotic infarction and mixed infarctions, there were too few cases for the results to be significant, but the rate of reduction in relative risk was equivalent to that for lacunar infarction, suggesting a uniform effect among the different infarct types. In cases of lacunar infarction, it is considered that there is a high risk of hemorrhagic events caused by arteriolosclerosis associated with high blood pressure, and this has triggered much debate concerning the safety of antiplatelet drug administration. However, the CSPS also presented evidence that cilostazol is an effective treatment for lacunar infarction, indicating that cilostazol administration should be considered once blood pressure has been thoroughly controlled. A CSPS subanalysis also showed that cilostazol has an excellent prevention rate of secondary cerebral infarction due to diabetic complications.

The South Korean Trial of Cilostazol in Symptomatic Intracranial Arterial Stenosis (TOSS) compared the effects of aspirin, both alone and in combination with cilostazol, on symptomatic intracranial arterial stenotic lesions. In this study, the combined administration of aspirin with cilostazol had significantly greater preventative effects on the progression of the lesions than aspirin alone [11].

Often, patients who have developed cerebral infarction also have multiple risk factors, and thus, it is vital to balance the management of such factors. Patients with chronic cerebral infarction show hemorrheologic abnormalities and decreased blood flow in an extensive area around the lesion site [15]. It is believed that such a clinical condition is linked to impairments in brain function and the aggravation of symptoms. Two of the main reported causes of secondary cerebral ischemia are decreased cerebral blood flow during rest and decreased vascular reserve [16]. Cilostazol, which not only has an antiplatelet effect but also acts as a vasodilator, therefore is a drug with the potential to address both issues simultaneously.

The present study confirmed that cerebral blood flow increased in each ROI, including the cerebral hemispheres, following cilostazol administration; this might be attributed to the arteriolar vasodilatory effect of cilostazol [5]. This arteriolar vasodilatory effect, in turn, was attributed to a pharmacological effect, which is independent of the vasodilatory effects of vascular endothelial nitric oxide (NO) or prostaglandin [5]. There is empirical evidence that administering cyclic adenosine monophosphate (cAMP) via the internal carotid artery increases cerebral blood flow [17], and the present study also demonstrated that cilostazol administration elevated arteriolar cAMP concentration and improved cerebral blood flow. Cilostazol is potentially useful for the treatment of cognitive impairment in post-stroke patients due to this accompanying increase in cerebral blood flow [18]. Choi [19] and Lee et al. [20] studied the neuroprotective action of cilostazol in animal models of cerebral infarction and attributed anti-apoptosis, anti-inflammatory, and antioxidant effects to it, as well as suggesting that cilostazol contributes to the maintenance of physiological functions. These findings suggest that cilostazol might also be involved in nerve regeneration, which will prompt further investigation of this aspect of its effects. In this study, two patients were investigated for physiological function, based on the performance of ADLs and cognitive function, following cilostazol administration. Both patients showed improvements according to the Barthel Index and based on MMSE scores. From the perspective of “pharmacological rehabilitation,” which maximizes the effects of rehabilitation by the strategic use of drugs, the present findings indicate that cilostazol has the potential to enhance rehabilitation in combination with other methods. The mechanism behind this effect of cilostazol is not properly understood, but the improvement in cerebral blood flow it produces is likely to be a contributing factor. Since continuous rehabilitation may have a cumulative effect on patients' functional recovery, a randomized controlled trial is planned in the future based on the findings from this report. The effects of cilostazol on the chronic after-effects of stroke will require further investigation in a larger sample of patients.

## Conclusions

Cilostazol exhibited a vasodilatory effect when administered to two patients with chronic atherothrombotic cerebral infarction. Following cilostazol administration, in both patients, there was a good enhancement in cerebral blood flow and there was a strong rehabilitation-enhancing effect. In terms of pharmacological rehabilitation, it is important to consider administering cilostazol to patients with chronic cerebral infarcts, either alone or in combination with another drug, because of the anticipated improvement in cerebral blood flow.
